# Increased posterior tibial slope in patients with anterior cruciate ligament‐deficient knees compared to knees with an intact anterior cruciate ligament and a degenerative medial meniscus tear: A radiographic comparative study

**DOI:** 10.1002/jeo2.70598

**Published:** 2025-12-17

**Authors:** Shintaro Onishi, Christophe Jacquet, Hiroshi Nakayama, Faisal M. Alfayyadh, Alexander J. Hoffer, Matthieu Ollivier

**Affiliations:** ^1^ Aix‐Marseille University, APHM, CNRS, ISM, Sainte‐Marguerite Hospital, Institute for Locomotion Marseille France; ^2^ Department of Orthopedic Surgery Hyogo Medical University Nishinomiya Japan; ^3^ Department of Orthopaedics University of British Columbia Vancouver Canada

**Keywords:** anterior cruciate ligament, degenerative meniscus tear, knee, medial meniscus tear, posterior tibial slope

## Abstract

**Purpose:**

The aim of this study was to quantify the difference in posterior tibial slope (PTS) in patients with anterior cruciate ligament (ACL) deficiency compared to those with a degenerative medial meniscus posterior horn tear (dMMPHT). We hypothesised that patients with ACL deficiency would have a greater PTS compared to patients with dMMPHT.

**Methods:**

All consecutive patients diagnosed with ACL deficiency (ACL group) or a dMMPHT with an intact ACL (MM group) from 2016 to 2022 at a single centre were reviewed. PTS was measured using the medial tibial plateau and tibial mechanical axis on standard lateral knee radiographs. Linear regression analysis was completed to assess the correlation between PTS and age in both groups. Further sub‐group analysis was undertaken according to age and sex of the study population.

**Results:**

In total, 294 patients in the ACL group and 250 patients in the MM group were analysed. Age differed between the groups (ACL group: 29.5 ± 10.4 years vs. MM group: 38.7 ± 8.5 years, *p* < 0.001). Radiological evaluation demonstrated increased mean PTS in the ACL group compared to the MM group (10.1° ± 3.0 vs. 6.7° ± 2.6, *p* < 0.001). Regression analysis showed no significant correlation between the PTS and age in each group. When patients were subdivided into those younger than 30 years of age and those 30 years or older, there was no significant difference in the PTS in the ACL group. In contrast, younger patients had a steeper PTS compared to those 30 years or older in the MM group (7.5° ± 1.9 vs. 6.5° ± 2.7, *p* = 0.005).

**Conclusions:**

Patients with an ACL tear had a significantly higher PTS on standard lateral knee radiographs compared to those with dMMPHTs and a normal ACL. Elevated PTS may be a risk factor for ACL rupture.

**Level of Evidence:**

Level III, retrospective cohort study.

**Abbreviations**
ACL: anterior cruciate ligament
ACLR: anterior cruciate ligament reconstruction
ATT: anterior tibial translation
dMMPHT: degenerative medial meniscus posterior horn tear
HFT: horizontal flap tears
HKA: hip‐knee‐ankle angle
MM: medial meniscus
MRI: magnetic resonance imaging
PTS: posterior tibial slope

## INTRODUCTION

Increased posterior tibial slope (PTS) has been postulated to play a significant role in anterior cruciate ligament (ACL) injuries. Previous biomechanical and clinical studies demonstrated a positive correlation between PTS and anterior tibial translation (ATT), which may result in elevated stress on native and reconstructed ACLs [2, 3, 10, 15, 19, 33, 43]. Further, increased PTS has been associated with a higher incidence of ACL injury and graft failure after ACL reconstruction (ACLR) [6, 7, 9, 20, 21, 30, 33, 40]. A PTS of 12° or greater on standard short knee radiographs is widely recognised as a risk factor for ACL rupture or graft failure after ACLR [30].

Recent studies have also identified an association between PTS and traumatic meniscal tears, particularly medial meniscus posterior root tears [8, 16, 18, 42]. Cadaveric studies have shown that elevated PTS leads to increased compression and anterior shear forces, and the posterior horn of the medial meniscus experienced the highest proportion of shear force during the weight‐bearing condition [24, 37]. Clinical studies have supported biomechanical models that show increased shear stress at the posterior medial meniscus with increased PTS [16, 26]. The normal aging process of the knee involves progressive structural and biochemical changes that predispose the menisci to degenerative tears [34]. Repetitive compressive and shear forces, even from daily activities, exacerbate intrasubstance degeneration, often culminating in horizontal cleavage or complex tear patterns [12]. While intrinsic biological aging drives meniscal weakening, biomechanical factors amplify vulnerability, making degenerative medial meniscus posterior horn tears (dMMPHT) a hallmark of the aging knee. Multiple studies have identified an association between increased PTS and posterior horn medial meniscus tears [16, 25, 39]. However, only Moon et al. previously completed a subgroup analysis based on the tear pattern, which showed no association between PTS and degenerative horizontal flap tears [25]. Therefore, the association between traumatic medial meniscal tears and increased PTS does not seem to exist with dMMPHTs. In addition, patients with isolated dMMPHT could be used as an alternative control group for patients without ACL injuries. However, no study has compared the PTS between patients with ACL deficiency to those with a dMMPHT and an intact ACL.

The aim of this study was to quantify the difference in PTS in patients with ACL deficiency compared to those with a dMMPHT and a rotationally stable knee. We hypothesised that patients with ACL deficiency would have a greater PTS compared to patients with dMMPHT and a rotationally stable knee.

## METHODS

### Patient selection and study design

A retrospective review of consecutive patients between 2016 and 2022 who were diagnosed with an ACL injury or dMMPHT at a single tertiary referral centre was performed. The inclusion criteria for the ACL injury group were a history of a traumatic knee injury with a diagnosed complete ACL rupture based on clinical findings and magnetic resonance imaging (MRI) confirmation. The inclusion criteria for the dMMPHT group were no history of trauma, with a diagnosed dMMPHT and no ACL abnormality based on clinical findings and MRI confirmation. Both these criteria were required to ensure the dMMPHT group was as close to a normal control knee as possible, while still ensuring the included patients had a full work‐up, including an MRI. A dMMPHT was defined as a horizontal cleavage or complex tear without extension into the meniscal body or root, with no extrusion. Exclusion criteria for both groups were subsequent ipsilateral or contralateral ACL injury, previous ligamentous injury regardless of surgery, and a lack of adequate data or proper radiographs. This study obtained ethics approval from the Institutional Review Board (PADS24‐171b‐dgr) and all patients provided written informed consent for study participation.

### Radiographic measurements and clinical variables

**Figure 1:**
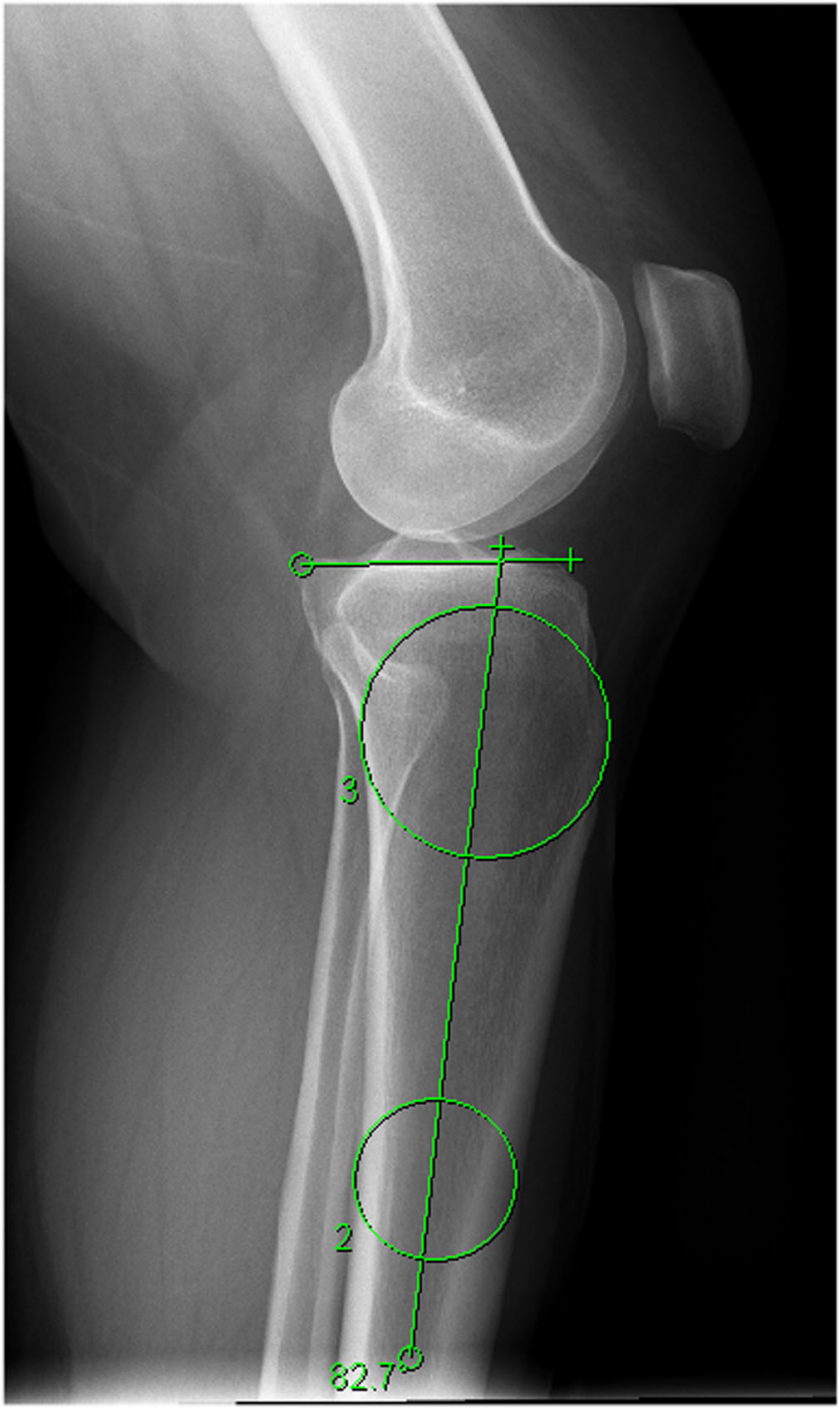
Measurement of PTS using a short knee radiograph. The PTS was calculated by the angle between a line tangential to the medial tibial plateau and the proximal anatomic axis of the tibia that was determined by a line passing through the mid‐cortical diameters of the tibia at a point 5 and 15 cm distal to the joint. PTS, posterior tibial slope.

Radiological and clinical data were retrospectively reviewed for acquisition and analysis. Demographic characteristics included age and sex. Radiological variables included PTS and the hip‐knee‐ankle angle (HKA). The PTS was measured on a standard lateral knee radiograph. Lateral radiographs were assessed for quality and considered appropriate if the cortical margins of the posterior femoral condyles were superimposed; otherwise, they were excluded from the analysis. The proximal anatomic axis of the tibia was determined by a line passing through the centres of two circles corresponding to the mid‐cortical diameters of the tibia at a point 5 and 15 cm distal to the joint [30, 40]. The PTS was obtained by measuring the angle between a line tangential to the medial tibial plateau and a line centred through the proximal anatomic axis of the tibia. (Figure 1) A fellowship‐trained musculoskeletal radiologist and knee subspecialized orthopaedic surgeon independently reviewed the blinded radiographs and measured the PTS twice 3 weeks apart. The accuracy of PTS measurements was evaluated using the intraclass correlation coefficient for intra‐ and interobserver reliability.

### Statistical analysis

Statistical analysis was performed using SPSS™ 12.0 (IBM Corporation, Somers, NY, USA). The Shapiro–Wilk test was used to determine if the data were normally distributed. Continuous variables were assessed with an unpaired *t*‐test for parametric data and a Mann–Whitney *U* test for non‐parametric data. Categorical variables were assessed with a chi‐squared test. Based on previous literature, a PTS cut‐off of 12° was set and the proportion of each group above the PTS cutoff was compared [30]. Linear regression analysis using the Pearson correlation coefficient was completed to test if sex and age significantly predicted PTS in both groups. Subgroup analysis by age and sex was completed. Regarding age, each group was divided into individuals younger than 30 and those aged 30 or older, as degenerative medial meniscus tears are rare in individuals under 30 [29]. Significance was set at *p* < 0.05 with a confidence interval of 95%, and all data are presented as mean and standard deviation.

A two‐tailed post hoc power analysis to assess achieved power was conducted using a sample size calculator (G*Power, version 3.1.9.6; Franz Faul, Universität Kiel) to compare the PTS between the ACL group and dMMPHT group. The study sample size of 294 in ACL group and 250 in dMMPHT group achieved 99% statistical power with an α of 0.05.

## RESULTS

**Figure 2:**
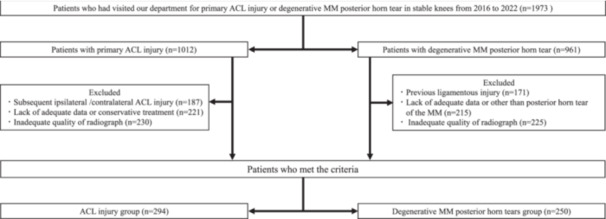
Flowchart of the patient selection process. ACL, anterior cruciate ligament; MM, medial meniscus.

**Figure 3:**
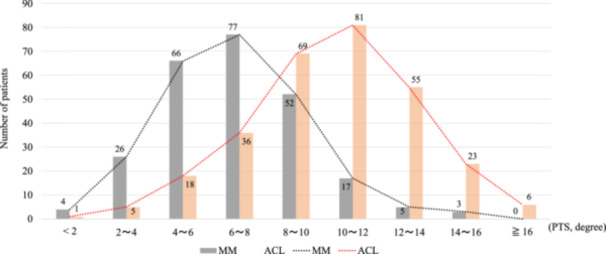
Histogram of PTS between patients with unilateral primary ACL and isolated degenerative MM posterior horn tear. ACL, anterior cruciate ligament; MM, medial meniscus; PTS, posterior tibial slope.

A total of 1973 consecutive patients were screened during the study collection dates. Of those, 544 patients were included in the final analysis (Figure 2). The ACL injury group was younger than the dMMPHT group (29.5 ± 10.4 vs 38.7 ± 8.5, *p* < 0.001). Patient demographics are summarised in Table 1. The mean PTS was higher in the ACL injury group compared to the dMMPHT group (10.1° ± 3.0 vs. 6.7° ± 2.6, *p* < 0.001). In addition, the proportion of patients with a PTS of 12° or more was higher in ACL injury group (28.6% vs. 3.2%, p < 0.001; Table 2). Six patients (2.0%) had a PTS greater than 16° in the ACL injury group compared to none in dMMPHT group (Figure 3). All intra‐ and interclass correlation coefficients for PTS measurement on lateral knee radiographs were 0.929 (95% CI, 0.911–0.943) and 0.841 (95% CI, 0.803–0.872), respectively.

**Table 1:** Demographic data.

	ACL group (*n* = 294)	dMMPHT group (*n* = 250)	*p* value
Age (years)	29.5 ± 10.4 (15–72)	38.7 ± 8.5 (18–50)	<0.001
Male/female (female, %)	206/88 (29.9%)	143/107 (42.8%)	0.002
Follow up period (years)	2.3 ± 3.4	2.0 ± 3.6	0.501

*Note*: Values are expressed as mean and standard deviations, with ranges in parentheses.

Abbreviations: ACL, anterior cruciate ligament; dMMPHT, degenerative medial meniscus posterior horn tear.

**Table 2:** Comparison of radiographic parameters between the ACL group and the dMMPHT group.

	ACL group (*n* = 294)	dMMPHT group (*n* = 250)	*p* value
HKA	178.0 ± 1.5 (173.2–181.0)	178.0 ± 1.6 (172.4–180.0)	0.856
PTS	10.1 ± 3.0 (1.0–20.0)	6.7 ± 2.6 (1.0–14.7)	<0.001
PTS ≧ 12°(*n*, %)	84 (28.6%)	8 (3.2%)	<0.001

*Note*: Values are expressed as mean and standard deviations, with ranges in parentheses.

Abbreviations: ACL, anterior cruciate ligament; dMMPHT, degenerative medial meniscus posterior horn tear; HKA, hip‐knee‐ankle angle; PTS, posterior tibial slope.

A linear regression analysis revealed no relationship between PTS and age or sex (ACL group: *β* = –0.052, SE = 2.991, *p* = 0.380, dMMPHT group: *β* = –0.116, SE = 2.559, *p* = 0.068) (Table 3). When the ACL injury group was sub‐divided into those younger than 30 and 30 years and older, there was no significant difference in the HKA or PTS. However, in the dMMPHT group, patients below 30 years of age had a higher PTS compared to those 30 years and older (7.5° ± 1.9 vs. 6.5° ± 2.7, *p* = 0.005) (Table 4). Further male patients under 30 years of age had a higher PTS compared to males 30 years and older in the dMMPHT group (7.7° ± 1.9 vs. 6.6° ± 2.6, *p* = 0.015; Table 5).

**Table 3:** Multi‐variate correlation analysis among the PTS and age, sex in the ACL group and dMMPHT group.

Variable	β Coefficient	95% CI lower	95% CI upper	*p* value
ACL group				
Age (per year)	−0.015	−0.048	0.018	0.370
Sex	−0.404	−1.158	0.349	0.291
dMMPHT group				
Age (per year)	−0.032	−0.070	0.006	0.094
Sex	+0.360	−0.289	1.009	0.275

*Note*: Values are expressed with 95% CI.

Abbreviations: ACL, anterior cruciate ligament; CI, confidential interval; MM, medial meniscus; PTS, posterior tibial slope.

**Table 4:** Comparison of posterior tibial slope between the patients aged younger than 30 years and over 30 years in ACL group and dMMPHT group.

	ACL group (*n* = 294)			dMMPHT group (*n* = 250)		
	Younger than 30 years (*n* = 173)	30 years and older (*n* = 121)	*p* value	Younger than 30 years (*n* = 45)	30 years and older (*n* = 205)	*p* value
HKA	178.0 ± 3.1	178.0 ± 3.4	0.691	178.2 ± 2.0	178.0 ± 2.8	0.716
PTS	10.1 ± 3.0	10.1 ± 2.9	0.955	7.5 ± 1.9	6.5 ± 2.7	0.005
PTS≧12 (*n*, %)	52 (30.1%)	32 (26.4%)	0.500	0 (0%)	8 (3.9%)	0.178

*Note*: Values are expressed as mean and standard deviations.

Abbreviations: ACL, anterior cruciate ligament; dMMPHT, degenerative medial meniscus posterior horn tear; HKA, hip‐knee‐ankle angle; PTS, posterior tibial slope.

**Table 5:** Comparison of posterior tibial slope between the males and females in ACL group and dMMPHT group.

	ACL group (*n* = 294)			dMMPHT group (*n* = 250)		
	Male (*n* = 206)	Female (*n* = 88)	*p* value	Male (*n* = 143)	Female (*n* = 107)	*p* value
PTS	9.8 ± 3.1	10.2 ± 2.9	0.377	6.9 ± 2.6	6.4 ± 2.5	0.188
Younger than 30 years (*n*)	10.4 ± 2.8 (*n* = 118)	9.4 ± 3.3 (*n* = 55)	0.053	7.7 ± 1.9 (n = 30)	7.0 ± 1.9 (*n* = 15)	0.252
Over 30 years (*n*)	9.9 ± 2.9 (*n* = 88)	10.6 ± 2.9 (*n* = 33)	0.256	6.6 ± 2.7 (*n* = 113)	6.3 ± 2.6 (*n* = 92)	0.427
*p* value	0.077	0.235		0.015	0.249	

*Note*: Values are expressed as mean and standard deviations.

Abbreviations: ACL, anterior cruciate ligament; dMMPHT, degenerative medial meniscus posterior horn tear; PTS, posterior tibial slope.

## DISCUSSION

The most important finding from this study was that patients who sustained a traumatic ACL tear had a higher mean PTS compared to those with no history of trauma, a stable knee and a dMMPHT. The mean PTS in patients with a primary ACL injury was 10.1° ± 3.0 and 29% of the patients in the ACL injury group had a PTS of 12° or more compared with 3% of the patients in the dMMPHT group.

Several studies previously quantified the normal PTS range using radiographic, computed tomography, or MRI measurements. The normal medial and lateral PTS range from 4.9° to 6.9° and 4.7° to 5.3°, respectively [27, 32, 41]. However, the PTS threshold that confers increased risk for ACLR failure varies depending on the imaging modality and measurement method used [7, 9, 32, 41]. Further, there may be only a weak correlation between radiographic and MRI PTS measurements [9, 14]. In a systematic review and meta‐analysis of studies assessing the relationship of PTS and ACLR failure, Duerr et al. found a mean PTS range from 5.4° to 17.2° in the ACLR failure group compared to 4.8°–14.4° in the control group among 6 studies that measured PTS using radiographic measurement methods [7]. In contrast, among 10 studies that used MRI measurement methods, the mean PTS range in the ACLR failure group was 2.7°–11.4° compared to 2.1°–7.1° in the control group [7]. Despite the variation in thresholds, the current literature highlights a prevailing relationship between elevated PTS and increased risk of ACL injury [4, 7, 20, 30, 40]. The reported mean PTS of 10.1° in patients with ACL‐deficient knees using the radiographic proximal anatomic axis measurement method in this study coincided with previous literature [1, 2]. Similarly, the mean PTS of 6.7° in the dMMPHT group was also comparable to the PTS in non‐ACL‐injured knees in previous studies [27, 32, 41].

There is a dearth of high‐quality evidence regarding the effect of increased PTS on native ACL injury and long‐term outcomes after ACLR. A steeper PTS increases both ATT and the strain on the ACL [22, 23, 33]. Multiple studies reported an association between elevated PTS and native ACL injury, summarised in a systematic review and meta‐analysis of 68 studies by Bayer et al. [1, 11, 28, 36]. But no causal relationship between elevated PTS and ACL rupture has been proven to date. Similarly, in the context of ACLR, Shelbourne et al. reported that patients with a PTS of 10° or more had a higher rate of graft failure with a mean follow‐up period of 11.6 years [31]. Salmon et al. demonstrated that a PTS of 12° or more was the crucial risk factor for subsequent ACL injury with a follow‐up of 20 years [30]. In a recent systematic review of level II–IV evidence, Liu et al. found an association between PTS and ACLR failure in 15 of 20 included studies. However, only 2 of the 20 studies included were level II studies [20]. The current study adds important information about the relationship between ACL insufficiency and PTS in a rapidly evolving field that currently lacks definitive evidence or treatment guidelines.

The relationship between PTS and degenerative tears of the posterior horn of the medial meniscus is not well delineated [8, 16]. Multiple studies have identified an association between increased PTS and medial meniscal tears, but it is unclear how the relationship changes depending on the specific tear pattern, location, chronicity and size [5, 13, 26, 39]. Moon et al. attempted to qualify the relationship between PTS and different MMPHT patterns. In a subgroup analysis of horizontal flap tears (HFT), there was no correlation between the HFT subgroup and PTS [25]. The mean dMMPHT PTS of 6.7° ± 2.6° in the current study closely coincided with Moon et al.'s subgroup analysis of the mean HFT PTS (6.5° ± 3.2°) [25]. The variability in measurement techniques, control group characteristics, and reported control group PTS in the literature makes it difficult to assess how the mean dMMPHT PTS in this study compares to knees with no associated pathology [1, 7]. Since the dMMPHT group in this study had no history of trauma and were diagnosed with horizontal cleavage tears or complex pattern posterior horn tears with no abnormality of the ACL on MRI, it is likely that this group more closely resembled a control group of ‘normal’ knees rather than a group of traumatic, vertical longitudinal medial meniscal tears.

The selection of patients with dMMPHTs as the control group in this study was a conscious and pragmatic decision to provide the most robust methodological framework possible. Unlike cohorts with acute trauma or ambiguous ACL status, all participants in the dMMPHT group underwent a comprehensive diagnostic workup, including lateral radiographs and MRI, that confirmed both the absence of a traumatic mechanism and the integrity of the ACL. Critically, these patients had verified normal ACLs without stretching or partial tears, a distinction that minimised the confounding factors while comparing tibial morphology between ACL‐injured and ACL‐intact populations. Prior studies highlighted the interplay between partial ACL tears and meniscal injuries, as even subtle ACL insufficiency may predispose the meniscus to increased shear forces during pivoting or loading [17, 38]. For instance, partial ACL tears have been frequently associated with concurrent medial meniscus tears, likely due to altered knee kinematics and instability amplifying stress on the meniscus [35]. A steeper PTS is associated with ATT and ACL strain, potentially creating a bidirectional risk: traumatic ACL injuries may occur more readily in knees with elevated slopes, while partial ACL tears secondary to slope‐related strain could indirectly contribute to degenerative meniscal pathology over time. However, in this study, the dMMPHT group had verified ACL integrity on MRI, which isolated the role of PTS in degenerative meniscal failure, independent of ligamentous compromise. This addressed limitations in earlier studies where undiagnosed partial ACL injuries might have obscured the relationship between PTS and meniscal pathology. By contrasting traumatic ACL tears with degenerative meniscal tears in ACL‐intact knees, our findings outline PTS as an independent anatomical risk factor for ACL injury, while also suggesting its potential contribution to dMMPHTs in younger populations via altered joint mechanics, even in the absence of ligamentous insufficiency.

### Limitations

The retrospective nature of this study introduced potential for recall bias, selection bias and confounding. Second, potential factors influencing ACL injuries and dMMPHTs other than coronal and sagittal bony alignment, such as sports activities, general laxity, and joint line obliquity, were not analysed. Third, demographics such as age and sex between the groups were significantly different. Although previous literature has shown no association between PTS and age [41], several studies reported that female patients have higher PTS compared to male patients [33, 41]. In our analysis, there were no significant differences in PTS between sexes. Furthermore, even though the regression analysis showed no significant correlation between age and PTS in the ACL group, the heterogeneity of patient demographics may have resulted in selection bias. Fourth, there was a slight but significant difference between the patients under 30 years of age and those 30 years and older in the dMMPHT group. The observed difference may have been influenced by the small sample size. In addition, patients with dMMPHT should not be considered representative of a healthy population. Lastly, the radiographic measurements may have been influenced by factors such as knee joint rotation. Although we excluded radiographs of inadequate quality to minimise measurement errors, this led to the exclusion of a substantial number of patients, which may have resulted in selection bias. Therefore, further studies are needed to assess the influence of PTS in patients with ACL tears compared with healthy populations.

## CONCLUSIONS

Patients with an ACL tear had a significantly higher PTS on standard lateral knee radiographs compared to those with dMMPHTs, a stable knee, and a normal ACL. Elevated PTS may be a risk factor for ACL rupture.

## AUTHOR CONTRIBUTIONS

All authors have contributed to the design, content and writing of the manuscript. All authors read and approved the final manuscript.

## CONFLICT OF INTEREST STATEMENT

Matthieu Ollivier has received consulting fees from Newclip Technics, Arthrex and Stryker. Other authors have nothing to declare.

## DATA AVAILABILITY STATEMENT

The data that support the findings of this study are available from the corresponding author upon reasonable request.

## ETHICS STATEMENT

Ethical approval for this study was obtained from the Institutional Review Board in our institution (PADS24‐171b‐dgr). Informed consent was obtained from all individual participants included in the study.
